# ^18^F-Fluorodeoxyglucose Imaging for Assessing Cardiovascular Inflammation

**DOI:** 10.3390/diagnostics15050573

**Published:** 2025-02-27

**Authors:** Nagara Tamaki, Tadao Aikawa, Osamu Manabe

**Affiliations:** 1 Kyoto University of Medical Science, Kyoto 622-0041, Japan; 2 Department of Cardiovascular Biology and Medicine, Juntendo University Graduate School of Medicine, Tokyo 113-8421, Japan; tadao.aikawa@juntendo.ac.jp; 3 Department of Radiology, Jichi Medical University Saitama Medical Center, Saitama 330-0834, Japan; omanabe@jichi.ac.jp

**Keywords:** cardiac inflammation, FDG, PET, sarcoidosis, vasculitis

## Abstract

Cardiovascular inflammation has recently emerged as a critical issue across various cardiovascular diseases. Various non-invasive imaging modalities are applied for assessing cardiovascular inflammation. Positron emission tomography (PET) using ^18^F-fluorodeoxyglucose (FDG) is a valuable non-invasive imaging tool for identifying active cardiovascular inflammation. It is utilized in evaluating conditions, such as cardiac sarcoidosis, endocarditis, vasculitis, and unstable atherosclerosis. Furthermore, management of cardiovascular complications after aggressive cancer therapy has increasingly been required in cancer patients. FDG PET is considered a suitable approach not only for the assessment of tumor responses to cancer therapy, but also for early and accurate detection of cardiovascular complications. This review highlights the clinical value of FDG PET under appropriate patient preparation. The future perspectives of new molecular imaging tools for assessing active cardiovascular inflammation have been described.

## 1. Introduction

Cardiovascular inflammation has emerged as a critical factor in a broad spectrum of cardiovascular conditions, such as myocarditis, cardiac sarcoidosis, and unstable vascular plaques. Non-invasive imaging, including molecular imaging, has been developed for the diagnosis and clinical evaluation of cardiovascular inflammation [1,2]. Among them, positron emission tomography (PET) has been in focus as one of the major molecular imaging techniques in clinical settings. The most commonly used PET tracer, ^18^F-fluorodeoxyglucose (FDG), is a glucose analogue radiopharmaceutical in which a hydroxyl group on the normal glucose molecule is replaced by ^18^F, a radioactive isotope of fluorine. FDG PET has been extensively employed for tissue characterization and treatment planning in oncology [3], and its application has expanded to include the identification and assessment of active cardiovascular inflammation.

FDG PET is gaining recognition as a critical non-invasive tool for identifying active inflammatory lesions associated with cardiovascular diseases and evaluating the efficacy of anti-inflammatory therapies. Furthermore, it is instrumental in the early detection and management of cardiovascular complications following cancer treatment [1,2,3,4,5,6,7,8,9,10].

This review summarizes the unique characteristics of FDG PET in the cardiovascular field among other non-invasive imaging modalities.

## 2. Method

Various non-invasive imaging modalities are available for assessing cardiovascular inflammation, each with distinct advantages and limitations, as summarized in Table 1.

Ultrasound imaging is widely available, cost-effective, and repeatable, and it does not involve the use of ionizing radiation [1,2,10]. Due to its high spatial resolution, it permits the precise assessment of structural abnormalities in the cardiovascular system. In addition, color doppler ultrasound and contrast-enhanced ultrasound are valuable for the assessment of functional and tissue alterations. It is generally performed by an experienced sonographer using high-quality instruments. The main limitation of ultrasound imaging is that it cannot depict structures beneath bone or air, and therefore, it does not provide reliable information about the thoracic aorta, unless performed via a transesophageal approach. In addition, the acquisition of ultrasound images is operator-dependent, although studies on vascular ultrasound have shown high rates of inter-operator agreement.

Computed Tomography (CT) scans are well known for their speed and high spatial resolution, enabling a detailed anatomical visualization that is crucial for the assessment of disease states [1,2,11]. The ability to capture fine structural details quickly makes CT an indispensable tool in acute care settings. However, the use of iodinated contrast media, necessary for enhancing image contrast, poses risks, particularly for patients with renal impairment. These patients may experience further renal damage or other adverse effects from the contrast agent. Additionally, the intrinsic radiation exposure associated with CT scans is a concern, particularly in scenarios requiring repeated imaging.

CT is nicely suited to demonstrate pathological changes in the cardiac wall, as well as in large, deep blood vessels [11]. It is a widely available and reproducible technique with high spatial resolution. CT angiography allows for the simultaneous assessment of the myocardial wall, the lumen, and the affected vessels. It also permits the detection of coronary stenosis. On the other hand, it involves the use of ionizing radiation and carries a risk connected to the use of iodinated contrast material.

Magnetic resonance imaging (MRI) offers distinct advantages over CT, particularly with its lack of ionizing radiation, making it safer for repeated use over time [1,2,7,8]. MRI has superior soft tissue contrast, making it ideal for imaging inflammatory changes in soft tissues and organs with superior clarity. However, the presence of loose metallic foreign bodies in a patient is a contraindication for MRI due to safety concerns and potential image distortion. Metal implants can also create significant artifacts, which may obscure diagnostic information and interfere with image interpretation. Despite these limitations, MRI remains a valuable tool for the detailed evaluation of inflammatory diseases, without the risks associated with radiation.

PET/CT may integrate functional and anatomical data in a single session [4]. This modality is particularly useful in evaluating inflammatory diseases, as it can highlight areas of metabolic activity associated with inflammation. Importantly, PET/CT can be safely used in patients with renal failure, as it does not require iodinated contrast media. Nevertheless, the long acquisition times and exposure to ionizing radiation are significant drawbacks. Additionally, while PET/CT provides crucial functional information, its spatial resolution is less detailed compared to standalone CT, which can be a limitation in resolving fine anatomical details. Similarly, the recently introduced PET/MRI modality has a significant impact for evaluating the cardiovascular system [7,8]. While MRI may require a long acquisition time, simultaneous imaging for both PET and MRI is available for clinical settings. PET/MRI may hold promise for assessing various functional and molecular functions in evaluating cardiovascular inflammation.

**Figure 1:**
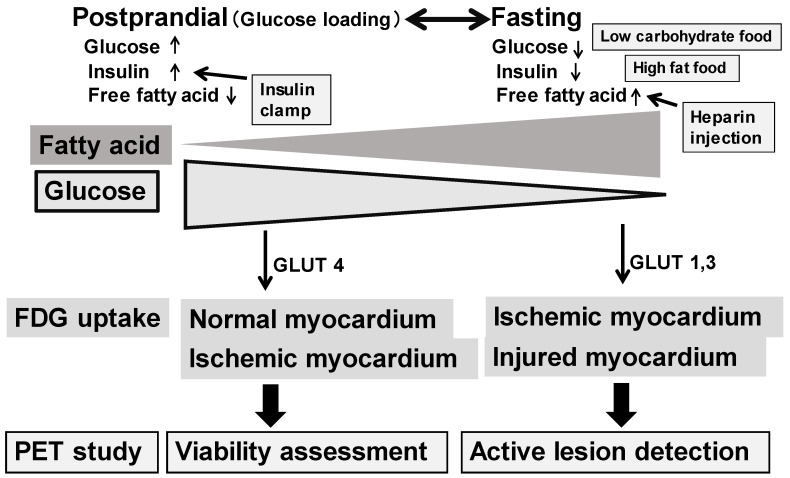
Differences in myocardial energy metabolism under postprandial and fasting conditions (**top**) and FDG uptake for a PET study (**bottom**). Glucose is a major energy source in the myocardium under high plasma glucose and insulin with low free fatty acid conditions. On the other hand, fatty acid is a major energy source under low plasma glucose and insulin with high free fatty acid conditions.

FDG uptake in the myocardium is dependent on a cardiac energy substrate, either glucose or fatty acids, depending on postprandial or long fasting conditions [12,13] (Figure 1). FDG PET is applicable for any patient. In addition, whole-body imaging is available once FDG is administered. Thus, such whole-body studies are commonly used in studies in oncology and generalized inflammatory diseases. Glucose is the major energy source in the myocardium under postprandial or glucose loading conditions. Under these conditions, FDG uptake is seen in both the normal and ischemic myocardium. In fasting conditions, on the contrary, glucose energy metabolism is suppressed in the normal myocardium. FDG uptake is seen only in the ischemic and/or injured myocardium. Therefore, patient preparation before FDG administration is quite important since FDG uptake in the myocardium is observed as either physiological uptake or abnormal active uptake, depending on the patient’s nutritional condition [12,13,14,15,16,17,18]. Our study indicated that plasma free fatty acid (FFA) was a suitable marker to predict the suppression of physiological FDG uptake in the myocardium [13]. FFA was a better marker for such a prediction than either plasma glucose or insulin under a >18 h long fasting condition with or without the use of heparin administration.

One of the classical applications of PET is the assessment of myocardial viability. FDG PET identifies glucose metabolism in the heart and thus identifies myocardial viability [19,20,21]. A region with preserved FDG uptake indicates the presence of viable myocardium. For this purpose, glucose administration with oral loading or an insulin–glucose clamp is applied. This allows the visualization of an increase in FDG uptake in both the normal and ischemic but viable myocardium, in comparison with no FDG uptake in the infarcted tissue. A myocardial viability study using FDG PET has a great clinical impact when predicting reversible dysfunction after revascularization and prognosis in patients with coronary artery disease and left ventricular dysfunction [19,20,21].

**Figure 2:**
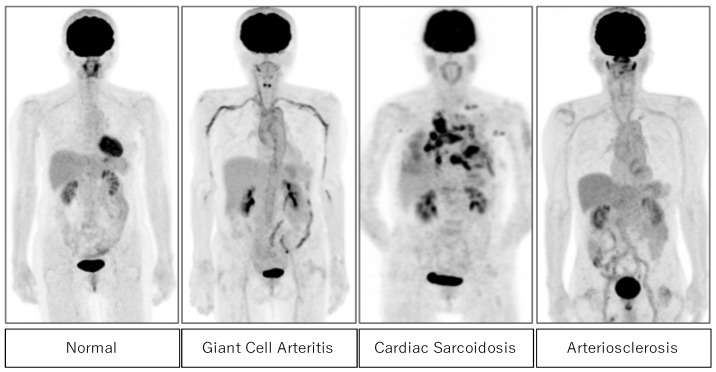
FDG uptake in the normal subject (**left**) and abnormal FDG uptake in active inflammatory lesions in patients with giant cell arteritis, cardiac sarcoidosis, and arteriosclerosis (**right**).

The activation of granulocytes and macrophages during inflammation enhances FDG uptake. Thus, FDG PET is useful for detecting active cardiovascular inflammation [22,23]. Figure 2 shows typical cases of positive FDG uptake in active inflammatory lesions in giant cell arteritis, cardiac sarcoidosis, and arteriosclerosis. High FDG uptake in large vessels is seen in giant cell arteritis and arteriosclerosis. In cardiac sarcoidosis, high FDG uptake in the myocardium and pulmonary nodules are frequently observed.

## 3. Results

### 3.1. Assessment of Cardiac Sarcoidosis

Sarcoidosis is a systemic granulomatous disorder of unknown cause [24]. While sarcoidosis in pulmonary and peripheral systems generally has a good prognosis, cardiac involvement has the potential for life-threatening consequences, such as an atrioventricular block, ventricular arrhythmias, congestive heart failure, and sudden death [25]. Accurate diagnosis and monitoring are critical for effective management.

It has been reported that about 5% of patients with sarcoidosis have cardiac signs/symptoms, but cardiac findings are more often found upon autopsy. In Western countries, no gender difference has been reported in the prevalence of cardiac involvement. In Japan, on the other hand, cardiac sarcoidosis is more common in middle-aged or older women [3].

Cardiac magnetic resonance imaging (MRI) and various radionuclide imaging, including FDG PET, have been added to the Japanese Ministry of Health and Welfare (JMHW) modified diagnostic criteria for cardiac sarcoidosis in 2006 [26] and 2016 [27]. The 2014 consensus statement by the Heart Rhythm Society (HRS) [28] indicated that histopathological diagnosis is required to reveal noncaseating granulomas from either an endomyocardial biopsy (EMB) or a surgical resection of the heart, which were previously described [24,25]. Clinical diagnosis requires concordance among electrocardiography, echocardiography, and cellular and molecular imaging, including late gadolinium enhancement on MRI and FDG PET.

**Figure 3:**
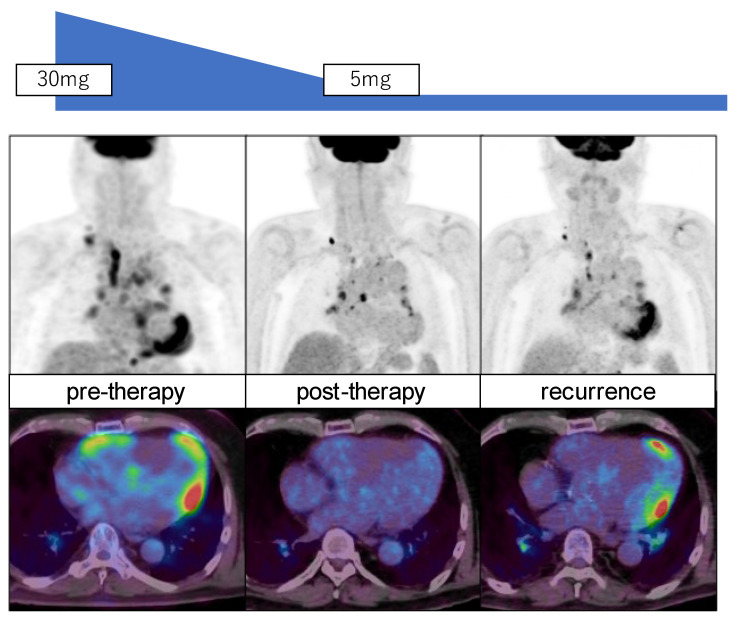
FDG PET MIP images (**top**) and transaxial images (**bottom**) of a patient with sarcoidosis before (**left**) and after steroid (prednisolone) therapy (**middle**) and in recurrent stage (**right**). Note significant reduction in FDG uptake in the myocardium and lymph nodes post-therapy with increased uptake in the myocardium with recurrence.

FDG PET is commonly used to assess the cellular infiltration of sarcoidosis in the myocardium. Since FDG uptake reflects the expression of glucose transporters, increased FDG uptake may reflect active inflammatory cells, such as macrophages, lymphocytes, and granulocytes. Thus, active sarcoid lesions may be nicely shown as focal FDG uptake in the myocardium as well as in other active sarcoid lesions in the body. Patient preparation is required, such as prolonged fasting, a low-carbohydrate diet, and/or a high-fat, high-protein diet to suppress physiological myocardial FDG uptake [29,30] (Figure 1). When plasma FFA is not high enough under regular fasting conditions, heparin administration may help to increase plasma FFA levels and suppress physiological FDG uptake in the myocardium. FDG PET, with an adequate preparation protocol, is ideal for detecting active myocardial lesions. When evaluating cardiac sarcoidosis, it is important to assess not only the extent of FDG uptake but also its location. The involvement of specific myocardial segments, particularly the basal to mid-anterior and mid-septal segments, is associated with higher event rates in patients with suspected cardiac sarcoidosis [31]. FDG PET is also valuable for assessing the response to anti-inflammatory therapy in patients with cardiac sarcoidosis [32,33,34,35,36]. Figure 3 illustrates a typical example indicating high FDG uptake in cardiovascular regions, which also disappeared after steroid therapy. The FDG uptake reappeared upon follow-up, suggesting a recurrence of the sarcoidosis. Thus, FDG PET is valuable for detecting active cardiovascular lesions and for monitoring treatment effects. Furthermore, FDG PET has potential for predicting patient outcomes [34,35].

A recent meta-analysis indicated that FDG PET had a slightly lower sensitivity than MRI, but both modalities showed a similar specificity for detecting cardiac sarcoidosis [36]. FDG PET may be valuable for identifying active inflammation with high FDG uptake but no fibrotic cardiac sarcoidosis. Thus, both FDG PET and MRI have important roles in diagnosing cardiac sarcoidosis and in its tissue characterization. FDG PET and MRI are useful for the early detection of cardiac sarcoidosis. Ohira et al. investigated the prevalence and characteristics of cardiac sarcoidosis in patients with biopsy-proven extracardiac sarcoidosis, comparing those with normal and abnormal 12-lead electrocardiography and transthoracic echocardiography findings [37]. They found that around 20% of patients with normal results on these tests still had cardiac sarcoidosis, emphasizing the importance of physicians remaining alert to the possibility of cardiac involvement even in the absence of conduction or structural abnormalities.

There are several clinical studies seeking to clarify the value of the semi-quantitative analysis of FDG uptake in active lesions associated with cardiac sarcoidosis. One recent report showed the value of follow-up with FDG PET in patients with active cardiac sarcoidosis [38]. After 12 months of prednisolone treatment, 80% of them showed a response with ≥70% metabolic reduction, but the remaining patients indicated either a poor response or recurrence. Further studies are needed to evaluate the long-term prognostic value of using FDG PET and MRI in patients with cardiac sarcoidosis.

### 3.2. Assessment of Endocarditis

Infective endocarditis is another severe cardiac inflammation, where FDG PET plays an important role in its diagnosis and management. Metastatic infections, embolic phenomena, or immune-mediated damage may cause considerable morbidity and mortality. In addition, infective endocarditis may often be associated with a prosthetic valve [39]. The diagnosis is based on the modified Duke criteria, including either microbiological evidence of infection by typical microorganisms and/or documentation of cardiac lesions by imaging techniques. Predisposing conditions, such as a fever, embolic vascular dissemination, immunological phenomena, and microbiological evidence have been considered as minor criteria [39,40].

Cardiac devices have been associated with increasing rates of device infections. Echocardiography is the first-line imaging test in patients with suspected endocarditis. It is challenging for patients with prosthetic valves. Cardiac CT and MRI are commonly applied for assessing endocarditis; however, severe artifacts from the metal device may cause limitations for clinical use. Additionally, abnormalities may not be specific for active infection. Therefore, they have an inherent limitation in accurate image analysis [39,40,41].

FDG PET/CT can identify septic embolism events, which is critical for patient management [41,42,43,44]. We indicated the importance of whole-body FDG PET in one interesting case showing septic regions in the finger of a patient with endocarditis [42]. FDG PET/CT may identify active inflammation in endocarditis earlier than structure abnormalities that can be assessed by echocardiography and CT.

In a meta-analysis of 13 studies involving 537 patients, PET/CT had a moderate sensitivity and specificity for the diagnosis of infective endocarditis [45]. However, the sensitivity improved when the evaluation of patients with suspected prosthetic valve endocarditis was selected, suggesting the potential value of FDG PET for use as an adjunctive diagnostic modality in challenging cases of possible infective endocarditis. Recent expert consensus recommendations from many European and American Societies support clinical values of FDG PET for the evaluation of suspected cardiovascular infection by increasing diagnostic accuracy, identifying extracardiac involvement, and assessing cardiac implanted device pockets, leads, and all portions of ventricular assist devices [46]. This may aid in key medical and surgical considerations.

### 3.3. Assessment of Vasculitis

Vasculitis is a group of disorders characterized by the inflammation of blood vessels, which can affect arteries, veins, or capillaries of various sizes. This inflammation can lead to vessel wall damage, narrowing, and even occlusion, disrupting blood flow and causing tissue damage. Vasculitis is classified based on the size of the affected vessels: large, medium, small, or variable vessel vasculitis, and vasculitis associated with systemic diseases or specific causes [47].

Large vessel vasculitis affects the largest arteries in the body, such as the aorta and its major branches. Large-vessel vasculitis is characterized by mononuclear and granulomatous infiltration of the vessel wall. Two key types include giant cell arteritis and Takayasu arteritis. Giant cell aortitis is more prevalent among Caucasians over the age of 50. It commonly affects the temporal arteries, leading to symptoms like headaches, jaw pain, and vision problems, and it can lead to serious complications, such as vision loss. Takayasu arteritis is mainly observed in female patients under the age of 40, with a predilection for individuals of Asian descent. The affected arteries in Takayasu arteritis are mainly the mesenteric, renal, and ilio-femoral arteries, while sparing the medium-sized cranial arteries. Takayasu arteritis has a greater propensity for causing severe focal stenotic lesions in the affected vessels than giant cell arteritis. In both large-vessel vasculitis, the inflammatory process may deteriorate the aortic wall, causing life-threatening vascular complications. Therefore, it is important to establish a diagnosis as early as possible using suitable biomarkers and imaging methods, including FDG PET [48,49].

Medium vessel vasculitis targets medium-sized arteries, which supply blood to specific organs. Polyarteritis Nodosa affects the main arteries of various organs, leading to organ ischemia (insufficient blood supply) and skin lesions. Kawasaki Disease, which primarily affects children, involves the coronary arteries and can lead to coronary artery aneurysms, along with a fever and other systemic symptoms.

Small vessel vasculitis involves the smallest blood vessels, including capillaries, venules, and arterioles. These conditions often affect the respiratory tract and kidneys, leading to sinusitis, lung nodules, and kidney disease. Another small vessel vasculitis, Churg–Strauss Syndrome (or Eosinophilic Granulomatosis with Polyangiitis, EGPA), is marked by asthma, high eosinophil levels, and involvement of the skin, nerves, and heart.

Variable vessel vasculitis can affect vessels of any size. For instance, Behçet’s Disease causes recurrent ulcers on mucous membranes (oral and genital) and uveitis (eye inflammation), while Cogan’s Syndrome affects the eyes and ears, leading to ocular and auditory disturbances.

Some types of vasculitis are associated with systemic diseases. For example, Rheumatoid Vasculitis occurs in severe cases of rheumatoid arthritis, typically involving small and medium vessels. Similarly, Lupus Vasculitis affects multiple organs in individuals with systemic lupus erythematosus.

Vasculitis is often characterized by immunologically induced inflammatory processes in vascular walls. The diagnosis and therapeutic monitoring of vasculitis are often challenging mainly due to the variable clinical, laboratory, and imaging manifestations. The choice of imaging method mainly depends on the size and localization of the affected vessels. Vessel lesions in small vessel vasculitis are usually below the radiologic detection limits. Vascular imaging can also help in the selection of the best biopsy point [50].

FDG PET imaging has recently been used for the accurate detection of vascular inflammation in the aortic wall [48,51,52,53]. Functional FDG PET, combined with anatomical CT angiography, may be of synergistic value for an optimal diagnosis, along with monitoring of disease activity and evaluating damage progression in large vessel vasculitis. There is a joint paper showing international recommendations and statements, based on the available evidence in the literature and the consensus of experts on FDG PET study, including patient preparation for an FDG PET/CT study, interpretation for the diagnosis, and follow-up of patients with suspected or diagnosed large vessel vasculitis [49]. FDG PET is considered a valuable tool for monitoring the treatment effects of this disease. Based on a review of the systematic literature, FDG PET showed a sensitivity of 77% and specificity of 71% for detecting relapsing/refractory disease in patients with large vasculitis [53]. In addition, a new recommendation for the use of imaging modalities, including FDG PET, has recently been reported in the diagnosis and management of patients with primary large vessel vasculitis [54]. FDG PET may hold an important role in distinguishing between large vessel aortitis subtypes, evaluating disease distribution, and detecting extracranial involvement in patients with cranial giant cell arteritis or polymyalgia phenotypes [55].

### 3.4. Assessment of Atherosclerosis

Atherosclerosis is a chronic condition characterized by arterial stiffening due to the buildup of cholesterol plaques on vessel walls. The progressive enlargement of these plaques may lead to peripheral artery disease, ischemic stroke, coronary artery disease, and acute myocardial infarctions.

Hypercholesterolemia with the deposition and oxidation of low-density lipoproteins in the arterial vessel wall may be responsible for the initiation and progression of atherogenesis. The migration, infiltration, and death of inflammatory monocytes/macrophages in the arterial wall may ultimately lead to the formation of vulnerable atherosclerotic plaques at a high risk for causing fatal cardiovascular events [56,57]. In addition, systemic inflammation, such as inflammatory or autoimmune diseases, may also accelerate the development of atherosclerosis, independent of lipid levels.

Non-invasive imaging is becoming more essential to investigate the role of inflammation in cardiovascular diseases. Conventional imaging modalities, including ultrasonography, CT, and MRI angiography, are widely used clinically to visualize large symptomatic plaques but are limited in their ability to assess the early stages of atherosclerosis [58,59]. Atherosclerotic plaques are another target for PET to identify microscopic inflammation. Molecular imaging using FDG and/or ^18^F-sodium fluoride (NAF) targeting microcalcification is clinically used in combination with high resolution CT or MRI [60,61]. The most used diagnostic signs are halo sign in ultrasound studies and visual positive uptake on FDG PET, which are often seen not only in large vessel vasculitis but also in atherosclerosis. When visually scoring FDG uptake intensity and patterns, FDG PET/CT attained 95% specificity for diagnosing LVV against an atherosclerotic control group [62].

These imaging methods are applied for assessing carotid plaques and even coronary plaques. A whole-body PET may identify unpredictable atherosclerotic plaques and even embolic lesions after endocarditis [63,64]. FDG vascular uptake is reflective of functional changes in the arterial vessel wall, which may predict structural changes related to the progression of atherosclerosis. FDG can identify unstable (vulnerable) plaques in the carotid and other vascular regions [65,66]. Higher FDG uptake is more often observed in unstable plaques with active inflammation than in stable plaques. Thus, FDG PET holds promise to identify plaque vulnerability. Furthermore, this study suggests that the early targeting of vascular inflammation with appropriate therapeutic regimens may slow down the formation of high-risk, vulnerable plaques [56,57].

### 3.5. Assessment in Cardio-Oncology

**Figure 4:**
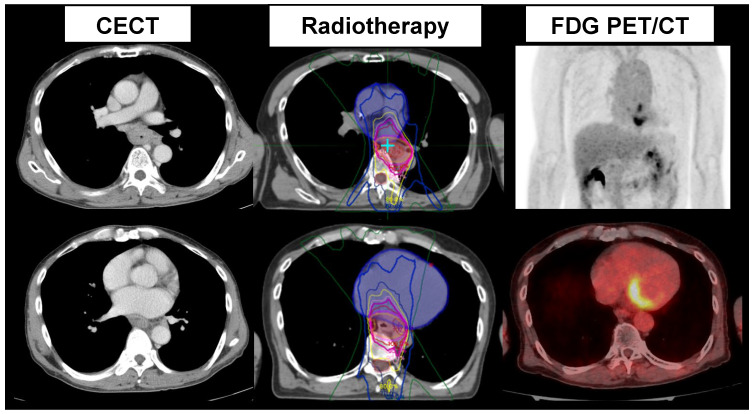
Contrast-enhanced CT (CECT) (**left**), radiation therapy planning (**middle**), and FDG PET/CT (**right**) after radiation therapy in a patient with esophageal cancer. High FDG uptake in the left atrium is noted, suggesting acute myocarditis after radiation therapy.

Cancer and cardiovascular disease are the leading causes of death in most developed countries. Cancer and cardiovascular disease are closely related with each other, particularly in older generations from both scientific and clinical perspectives. Recent advances in cancer treatment have improved patient outcomes. On the other hand, the management of cardiovascular complications has increasingly been required. Both cardiologists and oncologists should be cautious and exchange ideas for improvements in cancer patient care and ways to minimize cardiac dysfunction in this new field, termed “cardio-oncology” [67,68,69,70,71,72]. In addition, radiologists should play important roles in suitable image selection and in interpretations for assessing possible cardiovascular complications. Furthermore, radiation specialists should provide appropriate radiotherapy planning with reduced radiation doses to the cardiovascular system. Radiotherapy has recently been applied more often for cancer treatment. Radiotherapy is well known to have significant cardiovascular complications, such as pericarditis, and long-term complications, such as restrictive or constrictive pericarditis (Figure 4). Cardiovascular imaging has played a key role in the non-invasive assessment of cardiovascular alterations, complementary to biomarkers and clinical assessment. Cardiovascular imaging should also play an important role in suitable radiation planning, allowing for higher doses in the cancer tissue and minimal doses in the surrounding normal tissue [71,72].

Secondary large vessel vasculitis should also be considered. Extensive chemotherapy for cancer treatment, particularly with human epidermal growth factor receptor 2 (HER2) inhibitors and immune checkpoint inhibitors, may cause severe cardiovascular inflammation [67,68]. In addition, radiation therapy for esophageal cancer or breast cancer may possibly cause cardiovascular dysfunction [67,68]. Suitable therapies have significantly improved both survival and long-term outcomes in many cancer entities. On the other hand, such improvements in cancer outcomes are often accompanied by increasingly relevant therapy-related cardiovascular toxicity. Molecular imaging using MRI and nuclear imaging are used for assessing tissue function before and after cancer therapy from molecular perspectives. FDG PET is most commonly applied for the detection and extension of cancers, and it has an important role in providing suitable cancer management and treatment monitoring [71,72,73,74]. Also, FDG PET is valuable for detecting active cardiovascular inflammation, particularly in the early stages of cancer therapy, and monitoring toxic effects [71,72]. Arterial thrombosis and myocardial infarction may sometimes be observed as cardiovascular toxicities after cancer therapy. FDG serves as a sensitive indicator of the metabolic shift (from fatty acid utilization to glucose utilization) in the myocardium, which occurs in the early stages of coronary artery disease [71].

Active cardiovascular inflammation may often be observed after cancer treatment, which may possibly cause fatal cardiovascular dysfunction. Particularly, FDG PET is considered an elegant approach for the simultaneous assessment of tumor responses to cancer therapy and early detection of possible cardiovascular involvement [74,75,76,77]. For this purpose, suitable patient preparation is required to reduce physiological FDG uptake in the myocardium.

## 4. Discussion

FDG PET offers unique advantages over other imaging modalities for the detection and management of cardiovascular inflammation. Its ability to identify active lesions and evaluate therapeutic responses is rooted in its sensitivity to enhanced glucose metabolism in inflammatory cells. Proper patient preparation remains critical to minimizing physiological uptake and ensuring accurate lesion characterization. FDG PET has been used to detect active lesions and assess the response to anti-inflammatory therapy in patients with cardiac sarcoidosis. FDG PET has also been used to identify other instances of active cardiovascular inflammation, such as endocarditis, aortitis, and unstable atherosclerosis. Furthermore, FDG PET may be applied not only to assess responses to cancer therapy but also to detect possible cardiovascular involvement after cancer therapies, as shown in Figure 4. This review summarizes the clinical value of FDG PET in identifying and assessing active cardiovascular inflammation.

In Japan, the adoption of FDG PET for cardiac sarcoidosis under national insurance guidelines has set a global precedent as of 2012, enhancing its recognition and utilization worldwide [26,27]. FDG PET’s utility extends beyond sarcoidosis, encompassing endocarditis, vasculitis, and atherosclerosis, where it provides critical insights into both disease activity and progression. The use of FDG PET in endocarditis has not been approved yet, but worldwide clinical experiences in endocarditis, particularly after cardiac device application, should be the focus of many cardiologists. While echocardiology and MRI are commonly applied in these patients, FDG PET has several advantages, including detecting active inflammation, and has no apparent device artifacts. In addition, FDG PET is a powerful whole-body imaging tool, useful not only for identifying active myocardial lesions but also for detecting metastatic and embolic lesions throughout the body [39,40,41,42,43,44,45].

FDG PET has a great advantage in lesion characterization based on the FDG uptake value. Active cardiac sarcoidosis, endocarditis, aortitis, and even unstable plaques can be identified. FDG PET has promising utility in predicting clinical outcomes and assessing treatment responses based on the correlation between reductions in FDG uptake and improved disease control, since FDG uptake may be semi-quantitatively analyzed.

Most reports applied a combined analysis of structural and molecular changes using a PET/CT system. MRI can also identify morphologic and functional features of plaque instability. In addition, MRI permits the precise assessment of molecular function with superior soft tissue contrast, as described before. Thus, MRI has recently been applied for assessing cardiovascular inflammation in combination with FDG PET. MRI and PET provide complementary clinical information in the diagnosis of cardiovascular inflammation. More recently, the PET/MRI system has been made available in several centers. FDG PET/MRI has promise to provide information on various aspects of cardiovascular inflammation in vivo, such as edema and fibrosis, in combination with active inflammation [7,8].

On the other hand, PET has potential for assessing various new types of molecular information in the cardiovascular system. Fibroblast-activating protein (FAP) has been targeted by the ^68^Ga-labaled FAP inhibitor (FAPI), which is increasingly applied for assessing activated fibroblasts in various oncology patients [78]. FAPI PET has recently been applied for identifying acute myocardial infarctions and myocarditis with no need for the suppression of physiological myocardial uptake [77,79]. Another new molecular probe is ^18^F-sodium fluoride (NaF), which has the ability to detect molecular calcification. NaF PET has recently been used for atherosclerosis, and thus has the possible ability to identify unstable plaques [80]. NaF and FDG have been applied for identifying unstable coronary plaques using a suitable PET system [81].

Instrumental developments are also expected. Higher resolution PET systems have been introduced, which are cable of the precise assessment of the cardiovascular system, including vascular vulnerability. Quantitative assessment of FDG uptake may be more accurately performed. Such quantitative analyses should be applied for monitoring treatment responses based on the combined assessment of a refined structural and molecular basis [63,64,65,66]. More experiences with the use of FDG PET for analyzing cardiovascular inflammation will provide prognostic value for current imaging analysis methods in the near future.

## 5. Conclusions

FDG PET is valuable for detecting active cardiovascular inflammation mainly due to the enhanced glucose utilization associated with the activation of granulocytes and macrophages. This technique is used to identify active lesions and to assess responses to anti-inflammatory therapy in patients with various types of active cardiovascular inflammations. Furthermore, the management of cardiovascular complications after aggressive cancer therapy has increasingly been required in cancer patients. FDG PET serves a dual purpose by monitoring tumor responses to treatment while simultaneously identifying early cardiovascular complications. With appropriate patient preparation, FDG PET continues to demonstrate its value in improving diagnostic accuracy and informing clinical management across a range of cardiovascular and oncological conditions.

## Figures and Tables

**Table 1:** Features of non-invasive imaging techniques for assessing cardiovascular fields.

	Ultrasound	CT	MRI	FDG PET/CT
**Focus**	Sound waves Structure and flow analysis	X-way attenuation Structure and functional analysis	Proton density and echo time Edema and necrosis	Various molecular functions Glucose metabolism
**Patient advantage**	Easy to perform even at bedside No radiation	Very fast scan Evaluation of other organs	No or minimally invasive procedure No radiation	Both anatomical and functional information Safe in renal failure
**Patient disadvantage**	Relatively long acquisition time Operator dependent	Radiation associated with imaging Side effect by contrast media	Long acquisition time Claustrophobia due to smaller patient bore Contraindicated in patients with loose foreign metals High cost	Long acquisition time Radiation with radiopharmaceuticals High cost
**Imaging advantage**	High spatial resolution Real-time imaging Simultaneous assessment of cardiac function	High spatial resolution Can evaluate calcified plaques	Superior soft tissue imaging with excellent spatial resolution True multiplanar capability to image in any oblique plane	Providing functional and biological information Potential use of other molecular biomarkers
**Imaging disadvantage**	Limited use below bone or air Limited use for cardiac devices	Sub-optimal soft tissue imaging Lack of functional and biological information	MR image distortion Limited use for cardiac device	Limited spatial resolution Variable effect of thresholds or other criteria
